# Exploring the utility of cross-laboratory RAD-sequencing datasets for phylogenetic analysis

**DOI:** 10.1186/s13104-015-1261-2

**Published:** 2015-07-08

**Authors:** Serap Gonen, Stephen C Bishop, Ross D Houston

**Affiliations:** The Roslin Institute, University of Edinburgh, Midlothian, EH25 9RG Scotland, UK

**Keywords:** RAD-sequencing, Teleost phylogeny, Comparative mapping, Orthology

## Abstract

**Background:**

Restriction site-Associated DNA sequencing (RAD-Seq) is widely applied to generate genome-wide sequence and genetic marker datasets. RAD-Seq has been extensively utilised, both at the population level and across species, for example in the construction of phylogenetic trees. However, the consistency of RAD-Seq data generated in different laboratories, and the potential use of cross-species orthologous RAD loci in the estimation of genetic relationships, have not been widely investigated. This study describes the use of *Sbf*I RAD-Seq data for the estimation of evolutionary relationships amongst ten teleost fish species, using previously established phylogeny as a benchmark.

**Results:**

The number of orthologous *Sbf*I RAD loci identified decreased with increasing evolutionary distance between the species, with several thousand loci conserved across five salmonid species (divergence ~50 MY), and several hundred conserved across the more distantly related teleost species (divergence ~100–360 MY). The majority (>70%) of loci identified between the more distantly related species were genic in origin, suggesting that the bias of *Sbf*I towards genic regions is useful for identifying distant orthologs. Interspecific single nucleotide variants at each orthologous RAD locus were identified. Evolutionary relationships estimated using concatenated sequences of interspecific variants were congruent with previously published phylogenies, even for distantly (divergence up to ~360 MY) related species.

**Conclusion:**

Overall, this study has demonstrated that orthologous *Sbf*I RAD loci can be identified across closely and distantly related species. This has positive implications for the repeatability of *Sbf*I RAD-Seq and its potential to address research questions beyond the scope of the original studies. Furthermore, the concordance in tree topologies and relationships estimated in this study with published teleost phylogenies suggests that similar meta-datasets could be utilised in the prediction of evolutionary relationships across populations and species with readily available RAD-Seq datasets, but for which relationships remain uncharacterised.

**Electronic supplementary material:**

The online version of this article (doi:10.1186/s13104-015-1261-2) contains supplementary material, which is available to authorized users.

## Background

The recent advances in next-generation sequencing (NGS) technologies has meant that genotyping-by-sequencing technologies (such as RAD-Seq) are being utilised in both model and non-model organisms for a variety of applications (e.g. [1–9]). Genome-wide multi-locus data, such as those generated by RAD-Seq, are particularly advantageous for the estimation of evolutionary relationships. This is because unlike estimates obtained by comparing a single orthologous locus across multiple species, methods to address the problem of incomplete lineage sorting using multi-locus datasets are available [4, 10–14].

A particular advantage of RAD-Seq is that the inference of cross-population and cross-species orthologous loci is potentially simplified by the use of the same rare-cutting restriction enzyme (such as *Sbf*I) for the digestion of genomic DNA across all included individuals. Therefore, assuming no polymorphisms in the restriction site, the same genomic regions (i.e. homologous loci) can be sampled and concurrently sequenced across all individuals. The loss or gain of a restriction cut site due to the appearance of new mutations is likely to result in the identification of fewer orthologous RAD loci, particularly between more distantly related species. However, RAD-Seq protocols which involve digestion of genomic DNA using a single infrequent cutter followed by sonication of fragments are likely to be more robust to this issue than other RAD-like protocols (e.g. ddRAD [15]), where repeatable sampling of loci depends on the conservation of two restriction enzyme cut sites a certain distance apart on the genome. Overall, genetic relationships estimated using RAD data have been congruent with those seen in previously published literature (e.g. see Eaton et al. [4], Wang et al. [16]), suggesting that RAD data could prove useful in non-model taxa for which the evolutionary relationships are unknown.

Although RAD-Seq has been successfully applied in several phylogenetic studies (e.g. [4, 5, 16, 17]), these are typically based on sampling, sequencing and analysis by a single laboratory. The reproducibility of RAD loci across studies for the same species, and the ability to identify orthologous RAD loci across closely and distantly related species using cross-laboratory datasets, has not been widely investigated. In silico studies suggest that phylogenetic inference using RAD data may be restricted to relatively closely related species (<100 million years (MY) [18, 19]). Indeed, phylogenetic studies using empirical RAD-Seq datasets are restricted to the estimation of evolutionary relationships between closely related (<100 MY) species (e.g. [5, 20–22]). However, since RAD-Seq datasets from a wide variety of species and studies are now publically available, the utility of RAD-Seq for phylogeny estimation across more distantly related species can now be tested using experimentally-derived datasets. Additionally, while in silico phylogenetic studies have also investigated thresholds for inclusion of RAD loci with missing data (e.g. [23]), these thresholds have not been applied in real cross-laboratory datasets, where ‘missingness’ could arise for both technical as well as biological reasons.

Therefore, the overall aim of this study was to investigate the potential utility of cross-laboratory RAD-Seq data for estimation of phylogenetic relationships across closely and distantly related species, using ten species of teleost fish as an example. The specific aims of the study were to: (1) investigate the reproducibility of RAD data by aligning RAD sequences derived from different laboratories within-species; (2) investigate the performance of cross-laboratory RAD data in the inference of orthologous RAD loci and evolutionary relationships across species; and (3) investigate appropriate thresholds for inclusion of RAD loci for which there is missing data in some species.

## Results and discussion

Datasets generated by RAD-Seq using the *Sbf*I restriction enzyme were obtained from previously published studies for ten teleost fish species (five salmonid species and five non-salmonid teleost species). The five salmonid species included were: Atlantic salmon (*Salmo salar*), rainbow trout (*Onchorhynchus mykiss*), Chinook salmon (*Onchorhynchus tshawytscha*), sockeye salmon (*Onchorhynchus nerka*), and lake whitefish (*Coregonus clupeaformis*). The five non-salmonid species included were: three-spined stickleback (*Gasterosterus aculeatus*), Atlantic halibut (*Hippoglossus hippoglossus*), spotted gar (*Lepisosteus oculatus*), Baltic sea herring (*Clupea harengus*) and gudgeon (*Gnathopogon* sp.) (Table 1). The consensus RAD loci sequences (corresponding to the flanking sequences of the *Sbf*I cleavage sites), which were inferred based on the identification of RAD loci across multiple individuals within the population under investigation, were obtained for each study. Therefore, unlike studies which infer orthologous RAD loci across multiple taxa, insufficient sequencing depth at a given consensus RAD locus within a species is unlikely to be a problem in this study. In the case of Atlantic salmon and rainbow trout, data derived from two and four different studies respectively were utilised (Table 1). Within each dataset, the consensus sequences of the RAD loci were trimmed to 60 base pairs (bp) to be consistent across all studies (see “Methods”).

**Table 1:** Descriptions of the RAD sequences and the studies from which they were obtained

Species	Reference	Consensus sequence availability	Initial number of sequences	Sequence length (bp)	Post-processed number of sequences	Protocol and pipeline details				
						RAD-Seq library preparation protocol	Fragment size selection window (bp)	Sequencing platform	Sequence analysis pipeline	Minimum depth coverage per locus
Chinook salmon (*Oncorhynchus tshawytscha*)	Brieuc et al. [24]. *G3*, 4(3)	Online (SE)^e^	62,249	75	62,249	Baird et al. [25]	200–500	Illumina GAII/HiSeq	STACKS	Locus sequenced in 135 (85%) individuals
Sockeye salmon (*Oncorhynchus nerka*)	Everett et al. [26]. *BMC Genomics*, 13(521)	Provided by authors (SE)	64,613	60	64,613	Baird et al. [25] Etter et al. [27]	400–800	Illumina GAII/HiSeq	Custom-written Perl scripts, Bowtie, Novoalign	10 reads per allele per locus per individual
Rainbow trout (*Oncorhynchus mykiss*)	Hecht et al. [28]. *G3*, 2(9)	Provided by authors (SE)	12,073	67	32,027	Miller et al. [29] Baird et al. [25]	200–500	Illumina GAII/HiSeq 2000	Perl scripts from Miller et al. (2012), Novoalign	5 reads per locus per individual
	Hale et al. [30]. *G3*, 3(8)	Provided by authors (SE)	277,469	89		Miller et al. [31]	300–600	Illumina HiSeq	Perl scripts from Miller et al. (2012), Novocraft	5 reads per locus per individual
	Hohenlohe et al. [6]. *Molecular Ecology*, 22(11)	Online (PE)^f^	77,141	147–552^a^		Etter et al. [27]	330–400	Illumina HiSeq	STACKS	Locus sequenced in 1/60 (2%) individuals after pooling across individuals
	Miller et al. [31]. *Molecular Ecology*, 21(2)	Online (SE)^g^	40,649	68		Baird et al. [25] Hohenlohe et al. [6]	200–500	Illumina HiSeq	Custom-written Perl scripts, Novoalign	Locus sequenced in 3 individuals
Atlantic salmon (*Salmo salar*)	Gonen et al. [2]. *BMC Genomics*, 15(166)	Provided by authors (PE)	366,219	95	65,758	Etter et al. [27] with modifications from Houston et al. [1]	250–500	Illumina HiSeq 2000	RADtools, STACKS	500 reads per locus across 96 individuals
	Houston et al. [1]. *BMC Genomics*, 13(244)	Provided by authors (PE)	66,073^b^	95		Baird et al. [25] Etter et al. [27]	250–500	Illumina GAIIx/HiSeq 2000	RADtools	5 reads per allele per locus per individual
Lake whitefish (*Coregonus clupeaformis*)	Gagnaire et al. [8]. *Evolution*, 67(9)	Provided by authors (SE)	193,258	69	193,258	Baird et al. [25]	200–500	Illumina HiSeq 2000	STACKS	Locus is present in at least one mapping parent
Three-spined stickleback (*Gasterosterus aculeatus*)	Roesti et al. [32]. *Molecular Ecology*, 21(12)	Provided by authors (SE)	31,118^c^	64 or 138^d^	31,118	Baird et al. [25]	200–500	Illumina HiSeq 2000	Novoalign, SAMtools	12 reads per locus across 284 individuals
Atlantic halibut (*Hippoglossus hippoglossus*)	Palaiokostas et al. [33]. *BMC Genomics*, 14(566)	Provided by authors (SE)	83,678	96	83,678	Baird et al. [25] Etter et al. [27] with modifications from Houston et al. [1]	300–550	Illumina HiSeq 2000	STACKS	30 reads per locus per individual
Baltic sea herring (*Clupea harengus*)	Corander et al. [7] *Molecular Ecology*, 22(11)	Online (SE)^h^	63,742	95	63,742	Baird et al. [25] Hohenlohe et al. [6] Emerson et al. [34]	200–500	Illumina HiSeq 2000	FLORAGENEX unitag assembler v2.0, FLORAGENEX pipeline	5 reads per locus per individual
Spotted gar (*Lepisosteus oculatus*)	Amores et al. [35] *Genetics*, 188(4)	Provided by authors (SE)	64,483	75	64,483	Miller et al. [28] Baird et al. [25] Hohenlohe et al. [6]	200–500	Illumina GAIIx	STACKS	Locus sequenced in 85 (90%) individuals
Gudgeon (*Gnathopogon* sp.)	Kakioka et al. [36]. *BMC Genomics*, 14(32)	Online (SE)^i^	44,109	70	44,109	Etter et al. [27]	300–500	Illumina GAIIx/HiSeq 2000	STACKS	3 reads per locus per individual

*SE* single-end RAD-Seq, *PE* paired-end RAD-Seq.

^a^Paired-end RAD sequencing generated contigs of variable length.

^b^2 files from two families, sequence counts: 70,207 and 70,739. Subsequently combined into one file with 66,073 common sequences.

^c^46 files (one per individual). Sequence count range: 25,840 – 42,618. Subsequently combined into one file with 31,118 common sequences.

^d^Two separate sequencing studies were implemented, resulting in two different read lengths.

^e^ http://www.g3journal.org/lookup/ suppl/doi:10.1534/g3.113.009316/-/DC1.

^f^ http://datadryad.org/resource/  doi:10.5061/dryad.32b88

^g^ http://onlinelibrary.wiley.com/ doi:10.1111/j.1365-294X.2011.05305.x/

^h^doi:10.5061/dryad.jr56h.

^i^ http://www.biomedcentral.com/1471-2164/14/32/additional.

### Sharing of RAD loci across populations

To investigate RAD data reproducibility across populations (and studies) within species, orthologous RAD loci shared between the two different populations of Atlantic salmon, and between the four different populations of rainbow trout, were identified (Table 1; see Additional file 1 for details). A substantial overlap between RAD loci identified across studies was seen, with 99.5% of Atlantic salmon and 78.8% of rainbow trout sequences being shared across the different studies (percentages are given relative to the study with the fewest number of RAD loci). The higher percentage obtained across the two distinct Atlantic salmon populations may be partly due to the data originating from the same laboratory, and, therefore, more similar library preparation protocols and downstream bioinformatic analyses for data filtering. Overall, the results highlight the ability of RAD-Seq to consistently identify the same RAD loci across studies, despite inevitable technical variation in sample library preparation, sequencing platforms and downstream filtering pipelines. For example, subtle difference in RAD library preparation could affect the reproducibility of loci across studies (see Mastretta-Yanes et al. [37] for a review), including variations in the size selection window used after the sonication step of the protocol. Further, analysis pipelines with relatively strict thresholds for retaining homologous RAD loci across individuals (i.e. the population level consensus sequences utilised in this study), which are required for increased confidence SNP calling and genotyping within a population, could result in a decrease in the number of consensus RAD loci retained per species. This would reduce the number of informative loci available for relationship estimation.

### Sharing of RAD loci across species

The correct inference of sequence orthology across species is critical when estimating evolutionary relationships. As such, there is an abundance of literature on best practices for the inference of orthology, typically conditional on the availability of published reference genome sequences (e.g. see [38–40]). In the absence of well-assembled and annotated reference genomes for all included species, sequence similarity is thought to be a reliable way of inferring orthology [18], with higher power to detect orthologous relationships expected with longer sequences. However, the ability to detect orthologous loci based on sequence similarity decreases with increasing evolutionary distance due to the accumulation of mutations. This can be further complicated by major genomic rearrangements, such as the genome duplication that occurred in the Salmonidae [41, 42]. For RAD-Seq specifically, polymorphic variation in the restriction enzyme cut site, variation in methylation status of the locus (if the restriction enzyme is methylation sensitive), or genome rearrangements may further decrease the number of orthologous RAD loci identified [4, 20, 23, 43, 44]. Typical RAD-Seq analysis software (e.g. Stacks [45, 46] and PyRAD [47]) can readily identify homologous RAD loci within populations of individuals, but not necessarily across species when using consensus RAD loci sequences defined at the population level. One way of utilising these software in cross-laboratory and cross-species analyses would be to set the minimum coverage per locus (i.e. stack depth) to one within a given species and then to conduct comparisons across species to identify orthologous loci. In this study, cross-species orthologous loci were identified by pairwise and cross-species BLAST alignments, since BLAST alignment of sequences has been shown to reliably infer orthologous loci across species in the absence of reference genomes as utilised in similar studies (e.g. [26]).

To identify orthologous RAD loci using cross-laboratory datasets, pairwise alignments of consensus RAD sequences across the ten teleost species of varying levels of evolutionary relatedness was conducted. Firstly, pairwise alignments were clustered across salmonid species using strict alignment parameters (95% sequence identity, ≤2 base mismatch, minimum alignment length 50 bp) and, secondly, across all ten teleost species, using more relaxed parameters for alignment (85% sequence identity, ≤10 base mismatch, minimum alignment length 45 bp) (see “Methods” and Additional file 2 for further details).

A large number of orthologous loci were identified between the pairs of salmonid species, ranging from 6,500 to 16,000 (Additional file 3) when using strict alignment parameters. As expected, when alignment parameters were relaxed as described above, the number of putative orthologous RAD loci identified between pairs of salmonid species increased, ranging from 11,000 to 19,500 loci (Additional file 3). This may be due to the increased ability to infer orthology between RAD loci which lie within less conserved regions of the genome of these closely related species (divergence <50 MYA [48]), although a relaxation of alignment parameters is also likely to increase the number of false positive orthologies. Approximately half of the RAD loci were shared between pairs of *Oncorhynchus* species (rainbow trout, sockeye salmon, Chinook salmon). Sequence clustering based on these pairwise alignments identified a total of 3,050 loci with sequence present in all five salmonid species (‘clusters’) (Table 2). To investigate the effect of including RAD loci that are missing in some species, clusters with at least three sequences from three different salmonid species were identified. A total of 22,710 such RAD loci were identified, of which 78 were removed due to containing sequences which were assigned to multiple clusters (potential paralogous regions), leaving 22,632 clusters for further analysis (Table 2).

**Table 2:** Number of RAD locus clusters and interspecific variants identified for each analysis

Species	Parameters	Analysis pipeline	Minimum taxon coverage	Number of orthologous RAD loci	Number (%) of orthologous RAD loci in genes	Number of variants for relationship estimation	Range of missing interspecific variants in included species	Percentage of missing data in RAxML matrix
Salmonids	Strict	BLASTN	5	3,050	375 (12.3)	6,959	NA	0
Salmonids	Strict	BLASTN	≥3	22,632	1,407 (6.2)	39,890	3,135–21,480	25.09
All ten species	Relaxed	BLASTN	10	1	1 (100.0)	NA	NA	NA
All ten species	Relaxed	BLASTN	≥7	137	106 (77.4)	1,440	37–745	25.50
All ten species	Relaxed	BLASTN	≥5	452	321 (71.0)	4,094	371–2,881	36.75

In contrast, the number of shared RAD loci between pairs of the five distantly related (non-salmonid) species was much lower, with fewer than 500 (<2%) identified in most of the pairwise comparisons (using the ‘relaxed’ alignment parameters described above). For example, the number of orthologous loci in common between lake whitefish and Chinook salmon (~50 MY) was ~16,600, compared to ~300 loci common between Chinook salmon and spotted gar (~360 MY)—an ~55-fold reduction. Of the non-salmonid species pairwise comparisons, stickleback and Atlantic halibut contained the highest number of orthologous RAD loci (~2,700, 9%) as expected due to their closer evolutionary relationship (<100 MY) compared to any other pair of non-salmonid species in the study [42, 49, 50]. This is approximately a six-fold reduction in the number of shared RAD loci compared to lake whitefish and Chinook salmon, where the time since the last most recent common ancestor is almost half that of stickleback and Atlantic halibut.

Only a single RAD locus was identified in all ten species [predicted to occur within the gene coding for Transcription factor 7 (T cell specific, HMG box)]. Therefore, two inclusion thresholds were applied; (1) RAD loci with orthologous sequence in at least seven species (137 clusters); and (2) RAD loci with orthologous sequence in at least five species (4,945 clusters). To prevent bias in the estimation of evolutionary relationships, salmonid species-specific clusters were identified and removed (4,493 clusters), leaving 452 clusters with sequence for a minimum of five species including at least one non-salmonid.

### Identification of genic RAD loci

Given the higher degree of conservation of coding (i.e. genic) regions over evolutionary time [51, 52], it is plausible that the majority of orthologous RAD loci in the current study originate from coding regions. Previous studies have suggested that RAD loci obtained from *Sbf*I RAD-Seq analyses may be biased towards gene-rich regions of the genome, in part explained by the GC-rich nature of the *Sbf*I recognition sequence [2, 26, 35, 44, 53]. To test this hypothesis, all RAD loci consensus sequences were repeat-masked and aligned to a custom-made database of known fish gene nucleotide sequences, with significant alignment (E-value <1e^−5^) being evidence for a genic RAD locus (see “Methods”). In each of the individual salmonid species, approximately 2% of the RAD loci were identified as genic, and ~15% of the cross-species orthologous RAD clusters were predicted to originate from genes (Table 2). For each of the other (non-salmonid) teleost species individually, the percentage of genic RAD loci was higher (ranging from 4 to 50%), and >70% of cross-species orthologous RAD loci were identified as genic (Table 2). Alignment of genic loci across species identified very few (1–3 loci) which contained indels, suggesting high sequence conservation both at the nucleotide and amino acid level across species.

The lower ability to detect genic RAD loci within individual salmonid species (~2%) as compared to the other teleost species (up to 50%) in this study may be explained by the much larger genome sizes of the salmonid species (e.g. Atlantic salmon, ~3 GB; [54]) compared to the generally more compact genomes of the non-teleost species (e.g. stickleback, ~530 MB; [55]). The salmonid genome is known to be highly repetitive, (e.g. large number of transposable elements, repetitive tandem elements, etc.) [41, 42, 56–58]. This could mean that a larger proportion of the genome is non-coding, resulting in the identification of a lower proportion of genic RAD loci over the genome as a whole compared to species with compact, less repetitive genomes. Alternatively, the lower proportion of genic RAD loci predicted within the salmonid species may be attributed to the absence of salmonid gene sequences in the nucleotide database used for alignment, and the closer evolutionary relationship of the other teleost species with those in the database. In the case of stickleback, which has a high-quality, annotated reference genome and was included in the nucleotide database, ~50% of the RAD sequences were identified as genic. Based on the size of the stickleback genome (~530 MB; [55]) and the total length of known stickleback gene sequences (~192 MB; Ensembl 78, [59]), ~36% of the stickleback genome is estimated to be genic.

The large discrepancy in the proportion of cross-species orthologous genic RAD loci between salmonid (~15%) and non-salmonid (>70%) species may be due to the higher genome conservation (both coding and non-coding regions) across the salmonid species, due to their closer evolutionary relatedness. Overall, these results support the hypothesis that *Sbf*I RAD-Seq loci may be biased towards genic regions of the genome [26, 35, 44, 53], and this bias is useful for evolutionary and comparative genomics studies.

### Relationship estimation

**Figure 1:**
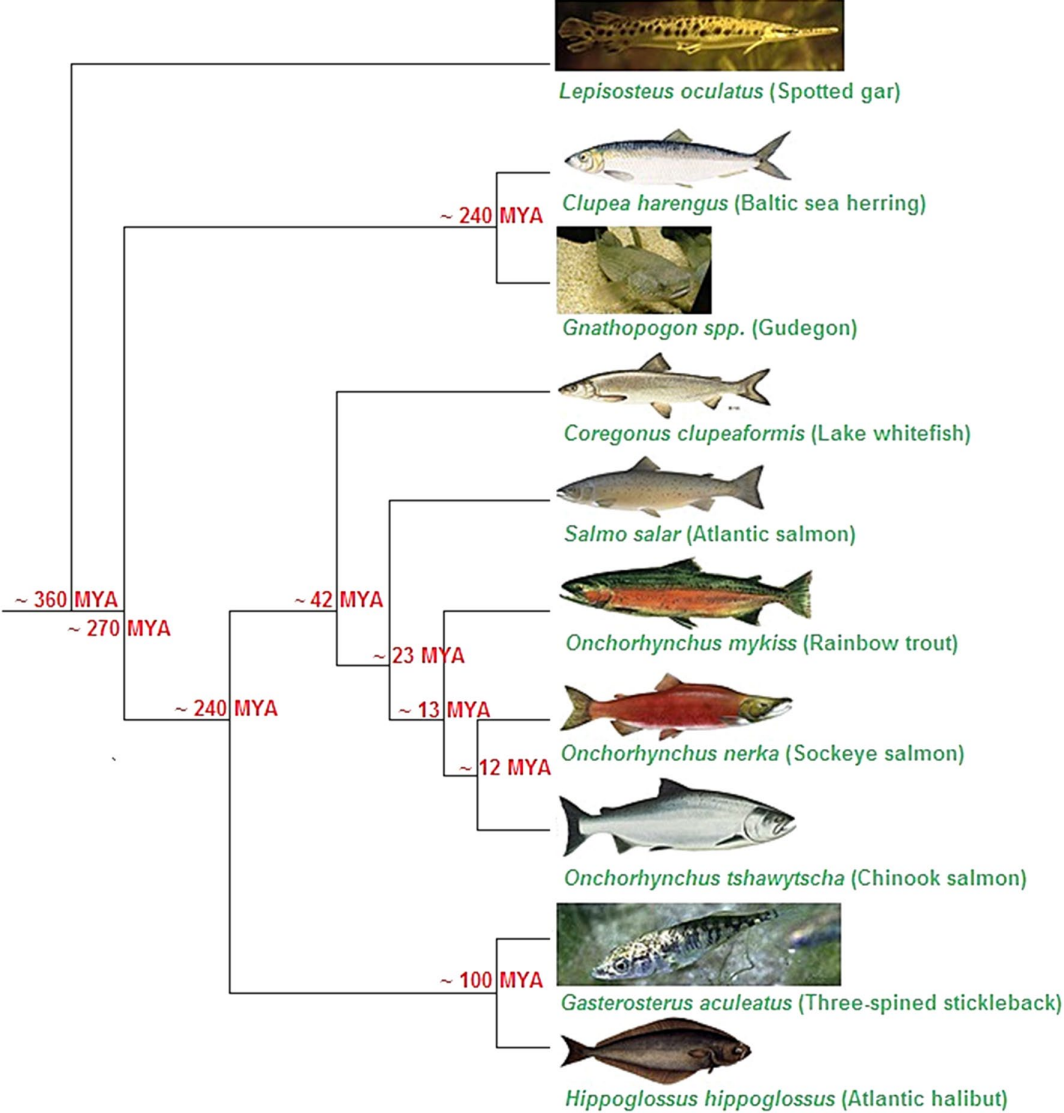
Expected evolutionary relationships as defined by Near et al. [49] and Shedko et al. [48]. Species images were taken from http://en.wikipedia.org/ or are published for open access use. Divergence times and branch lengths not drawn to scale. Divergence estimates for the non-salmonid teleost fish species were obtained from Near et al. [49], and divergence estimates for the salmonid species were obtained from Shedko et al. [48].

To our knowledge, the most comprehensive study of teleost phylogeny is that described in Near et al. [49] (232 fish species; nine coding sequences and fossil calibration times). Based on this phylogeny and the salmonid species relationships described in Shedko et al. [48], the expected relationships between the ten teleost species in the current study are given in Figure 1.

To test the utility of the cross-species and cross-laboratory RAD datasets in the construction of phylogenetic trees, multiple alignments of sequences within orthologous RAD clusters was conducted. This allowed the identification of interspecific single nucleotide variants, which were concatenated into a single sequence for each species and used to estimate evolutionary relationships (RAxML software; see Additional file 4 for RAxML parameters). RAxML input files used in all analyses are available at: doi:10.5061/dryad.bg6m0.

Whilst strict filtering thresholds applied in RAD-Seq studies often result in the removal of loci or individuals with excess missing data, recent simulation studies suggest that more relaxed thresholds could be favourable in resolving relationships [4, 23]. In the current study, a comparison was made between phylogenetic tree construction using stringent and more relaxed thresholds for RAD loci missingness across species.

Firstly, for estimating the phylogenetic relationships between the five salmonid species only, dataset 1 included RAD loci present in all five salmonid species (3,050 loci, 6,959 variants; Table 2), whilst dataset 2 included RAD loci present in at least three of the five salmonid species (22,632 loci, 39,890 variants; Table 2). Both datasets were able to recover the expected relationships between the five salmonid species (based on Shedko et al. [48]), with the three *Oncorhynchus* species forming a monophyletic group relative to Atlantic salmon and lake whitefish (all nodes >96% bootstrap support; Additional file 5, trees 1 and 2).

**Figure 2:**
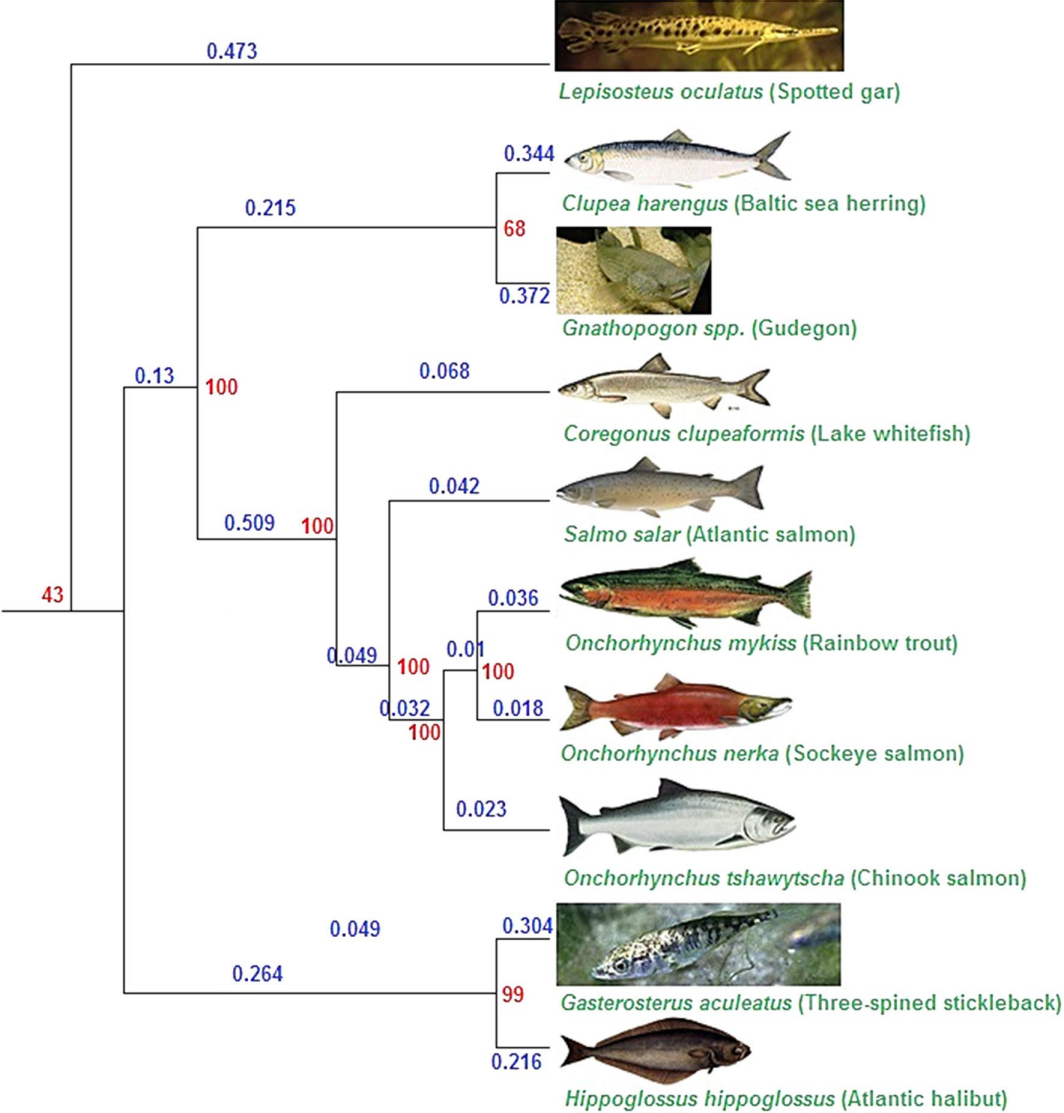
Example tree of all ten fish species obtained in this study using RAxML. Evolutionary relationships obtained using RAD data in this study were congruent with those of Near et al. [49] (teleost species) and Shedko et al. [48] (salmonid species) (Figure 1). Parameters—RAD loci present in at least five of ten species; 452 loci, 4,094 between-species variants. Branch lengths (given as percentages) estimated in RAxML are given along individual branches (in *blue*), and node bootstrap support values (1,000 bootstrap replicates) are given at individual nodes (in *red*). Branch lengths are not drawn to scale.

Likewise, across the ten teleost fish species, evolutionary relationships were estimated using variants derived from RAD loci common to at least seven of the ten species (137 loci, 1,440 variants; Table 2) and compared to the estimates using orthologous RAD clusters common to at least five of the ten species (452 loci, 4,094 variants; Table 2). Overall, tree topologies were consistent with previously published literature (Figures 1, 2; Additional file 5, trees 3 and 4). Monophyly of the Salmonidae and monophyly of the three *Onchorhynchus* species was predicted with 100% bootstrap support. Across both the salmonid and the teleost datasets, relaxing the threshold for inclusion of RAD loci in the analysis did not change estimated relationships or tree topology. Improvements in node support were also observed, for example, all salmonid species nodes were estimated with 100% support (vs. 98–100%) when the minimum taxon coverage at a RAD locus was reduced from seven to five of the ten species included (e.g. Additional file 5, trees 3 and 4). However, improvements in node support were not seen in all cases, for example, the node placing spotted gar as outgroup was not as strongly supported when the minimum taxon coverage was reduced (48–80%; Additional file 5, trees 3 and 4). Although bootstrap support is generally accepted as a reliable indicator of node accuracy, recent in silico studies suggest that this may not always be the case with RAD-Seq data [18]. Since true node support values obtained using empirical datasets are unknown, the accuracy of the reported bootstrap values cannot be quantified in this study.

Although tree topologies were generally consistent with published studies, there were some noteworthy differences. For example, in Figure 1 (phylogeny from Near et al. [49] and Shedko et al. [48]), the node connecting stickleback and Atlantic halibut is placed as the sister group to the salmonid species, whereas in Figure 2 (this study, loci with a minimum taxon coverage of 5), the node connecting the Baltic sea herring and gudgeon is placed as sister species to the salmonid lineage, with 100% bootstrap support. However, this was not seen with loci with a minimum taxon coverage of 7 (Additional file 5, tree 3). Recent simulation studies have suggested that the resolution of RAD data is low when estimating relationships between distantly related species (>100 MY) [18–20]. However, although monophyly of the *Onchorhynchus* species (<13 MY) were predicted with 100% node support, relationships between the species differed depending on the minimum taxon coverage per locus, as well as when using salmonid species specific loci vs loci across all included species (Additional file 5). This is contrary to the expectations of better estimates of relationships between closely related species using RAD datasets as suggested by simulation studies [18–20], and suggests that caution must be applied when interpreting both shallow and deeper evolutionary relationships using this method.

In some cases (for example in the branch separating the salmonid species from the other five teleost species; Additional file 5, trees 3 and 4), branch lengths estimated using loci with a minimum of five species with sequence were approximately double that estimated using loci with a minimum of seven species with sequence. Therefore, while minor variation in the thresholds for inclusion of RAD loci absent in some species is unlikely to affect estimation of evolutionary relationships, it could potentially bias the estimated divergence times between more distantly related species (not estimated in this study). Therefore, the thresholds for inclusion of RAD loci with missing data should be considered and tested before utilising RAD loci for estimating relationships between species.

## Conclusion

In this study, RAD-Seq datasets derived from different laboratories were utilised in the estimation of evolutionary relationships between ten teleost fish species. Within species and across populations, a large proportion of shared RAD loci were identified (78–100%), despite variation in laboratory techniques and bioinformatic pipelines. As expected, the number of orthologous RAD loci identified across species decreased as the evolutionary distance increased, ranging from ~3,000 between the most closely related salmonid species to ~450 between distantly related species. Multiple alignments of sequences within orthologous RAD loci allowed the identification of interspecific single nucleotide variants, which were used to estimate evolutionary relationships. These were consistent with previously published phylogenies, even across very distantly related species. Approximately 70% of the orthologous RAD loci used in the analysis of the ten teleost species were predicted to be genic, providing support for previous findings of a the bias of *Sbf*I RAD loci towards genic regions, which is likely to facilitate relationship estimation between distantly related species. Overall, this study has highlighted the potential utility of experimentally-derived cross-laboratory RAD-Seq datasets in the estimation of evolutionary relationships across closely and distantly related species.

## Methods

### Sequence data

In a typical population genetics RAD-Seq bioinformatic pipeline, sequence reads derived from the flanking regions of the restriction enzyme are collapsed into a single ‘RAD locus’ [25]. For each locus, sequence reads are aligned within and then across individuals, and a single ‘consensus sequence’ is generated. In the case that a particular nucleotide site is polymorphic in a given population, the consensus sequences will show the allele with the highest frequency (>50%). Single-end *Sbf*I RAD consensus sequences (i.e. both monomorphic and polymorphic consensus sequences) were obtained for Atlantic salmon (*Salmo salar*), rainbow trout (*Onchorhynchus mykiss*), three-spined stickleback (*Gasterosterus aculeatus*), gudgeon (*Gnathopogon* sp.), Chinook salmon (*Onchorhynchus tshawytscha*), sockeye salmon (*Onchorhynchus nerka*), spotted gar (*Lepisosteus oculatus*), lake whitefish (*Coregonus clupeaformis*), Baltic sea herring (*Clupea harengus*), and Atlantic halibut (*Hippoglossus hippoglossus*) (details specific for each study are given in Table 1). RAD-Seq studies using the *Sbf*I restriction enzyme were chosen since this is the most commonly used protocol within aquatic species, and, therefore, had the most publically available data.

For rainbow trout and Atlantic salmon, data from four and two different studies respectively were obtained. For stickleback, consensus RAD sequences were generated within individuals (N = 46) and aligned to the reference genome, and population-level consensus sequences were unavailable (Table 1). For each of these three fish species, a single file of common RAD loci was produced using BLASTN alignments of all sequences (95% identity, ≤2 base mismatch), where common RAD loci were defined if sequence for that locus was observed in more than a certain threshold number of populations/individuals (see Additional file 1).

### Data filtering, processing and characterisation

The consensus sequence files from each of the ten species were processed as follows. To avoid bias in alignment parameters due to differences in sequence lengths [60, 61], all sequences were trimmed to 60 bp (the shortest read length amongst the studies). To limit the misleading alignment of sequences to multiple regions due to genomic repetitive elements, low complexity sequences were masked using RepeatMasker [62] (parameters: -s; -lib; -gccalc). To minimise the effect of repeat sequences in potentially duplicated regions of the salmonid species genomes, the Atlantic salmon repetitive element database (http://web.uvic.ca/grasp/salmon_v1.6) was additionally utilised as a library within RepeatMasker.

To investigate the previously reported bias of *Sbf*I RAD-Seq to gene-rich regions of the genome [26, 35, 44, 53], trimmed and repeat-masked sequences for each of the ten species were individually aligned (TBLASTX; BLAST+ version 2.2.25+ ; [63]) to a custom-made database of nucleotide gene sequences. This database comprised gene sequences originating from Atlantic cod (*Gadus morhua*), puffer fish (*Takifugu rubripes*), medaka (*Oryzias latipes*), platyfish (*Xiphophorus maculatus*), spotted gar (*Lepisosteus oculatus*), three-spined stickleback (*Gasterosterus aculeatus*), Tetraodon (*Tetraodon nigroviridis*), tilapia (*Oreochromis niloticus*) and zebrafish (*Danio rerio*) (Ensembl 78 [59]). Alignment significance was taken at E-value <1e^−5^.

### Identification of cross-species orthologous RAD loci

To identify RAD loci conserved across species, pairwise BLASTN analyses of the trimmed and repeat-masked consensus RAD sequences were conducted (‘blastn’ alignment algorithm; BLAST+ version 2.2.25+ ; [63]). The most significant alignment for each sequence (i.e. ‘best hit’) was extracted. Two files of best hits were created: (1) within salmonid species only; and (2) across all ten species (including the salmonid species).

Best hit alignment files were quality-checked and filtered based on the following thresholds: (1) within salmonid species only, using ‘strict’ alignment parameters of ≥95% percentage identity, ≥50 bp alignment length and ≤2 base mismatches; and (2) between all ten species, using more ‘relaxed’ alignment parameters of ≥85% percentage identity, ≥45 bp alignment length and ≤10 base mismatches. The stricter alignment thresholds within salmonids were chosen in an attempt to differentiate between both orthologous and paralogous regions of the salmonid genomes. Alignment parameters remained constant within each analysis (rather than varying parameters according to the evolutionary distance between species) such that: (1) consistency in parameters across all pairwise alignments was maintained, in order to aid comparisons of the number of loci identified between species of differing relatedness; and (2) the identification of misleading alignments (for example between sequences corresponding to conserved regions of the same gene family rather than the same RAD locus) is minimised. To minimise multiple alignments of sequences within salmonid species due to the recent (~90 MYA; [58]) salmonid specific genome duplication [41, 42] or due to uncharacterised repetitive elements across all species, all pairwise alignments were further filtered to retain only unique alignments (i.e. where the subject sequence was the best hit to a single query sequence). Two files of pairwise best hits were created: (1) within salmonids; and (2) across all ten fish.

To identify orthologous RAD loci across groups of species of differing levels of evolutionary relatedness, pairwise alignments were clustered, first within the salmonid species only based on the strict pairwise alignments, and second, across all ten species, based on the relaxed alignment parameters. The clustering pipeline was implemented as follows (also see Additional file 2). Using the two files of filtered pairwise best hits, sequence clusters were inferred if RAD locus sequences across all included species all aligned to each other respectively as the most significant and unique match. To limit the effect of paralogous sequences on inferring clusters across the salmonids and unidentified repetitive elements across all species, clusters containing sequences which were assigned to multiple clusters were removed. Clusters containing more than one RAD locus sequence from a single species were removed.

To analyse the effect of incorporating RAD loci which were ‘absent’ for a given species (i.e. no ortholog identified in the available dataset), clusters were filtered using varying thresholds for sequence absence. Within the salmonid species strict analysis, clusters containing sequences from all five salmonid species and clusters containing sequences from at least three of the five salmonid species were retained. Across all ten species, only a single RAD locus cluster was identified. Therefore, downstream analyses were conducted using clusters with a minimum of seven sequences from at least seven different species or a minimum of five sequences from at least five different species. To prevent bias in the estimation of evolutionary relationships, these clusters were further filtered to remove salmonid species-specific clusters, i.e. clusters that contained sequences originating from salmonid species only. The proportion of clusters within genic regions of the genome was quantified, based on alignment to the custom-made fish nucleotide gene database, as described above.

### Reconstructing teleost fish phylogeny using RAD data

To test the utility of cross-laboratory RAD-Seq data to infer teleost species relationships, cross-species orthologous RAD locus clusters described above were used to construct phylogenetic trees. For each identified RAD locus cluster, sequences for each species within the cluster were extracted. If absence of a RAD locus for a given species was permitted (as in salmonid dataset 2 and all fish datasets 1 and 2), species with no sequence for that locus were assigned a string of 60 * ‘N’. Sequences within a cluster were aligned using the MUSCLE software (version 3.8.31 [64]), and the resulting alignments were investigated for the presence of between-species single nucleotide variants. Alleles for each variant for each species across all RAD loci were concatenated into a single sequence. Concatenated variant sequence files were converted into the PHYLIP format [65] for input into the RAxML software (version 8 [66]) (see Additional file 4 for details on RAxML parameters). RAxML employs a maximum likelihood based algorithm for phylogeny inference, and was chosen since it allows for correction of ascertainment bias which may arise when using variants for relationship estimation. RAxML was run using 1,000 bootstraps for all analyses.

## Additional files

Additional file 1: Inferring consensus RAD sequences within species. Additional file 2: Clustering of pairwise BLASTN alignments into cross-species orthologous RAD loci. Additional file 3: Number and percentage of shared RAD loci identified by pairwise BLASTN alignments. Additional file 4: Parameters for phylogenetic tree construction using RAxML. Additional file 5: Estimated phylogenetic relationships.
