# Supplementary material for: Exploring the utility of cross-laboratory RAD-sequencing datasets for phylogenetic analysis
Source: BMC Res Notes. 2015 Jul 8;8:299. doi: 10.1186/s13104-015-1261-2 (PMC4495686; doi:10.1186/s13104-015-1261-2)
Supplement: Additional file 1: — Inferring consensus RAD sequences within species. [file 13104_2015_1261_MOESM1_ESM.docx]

# Processing and combining consensus RAD sequences within species

## Rainbow trout

Four FASTA files from four different studies ([Hohenlohe *et al*, 2011](#_ENREF_6); [Hecht *et al*, 2012](#_ENREF_4); [Hale *et al*, 2013](#_ENREF_3); [Hecht *et al*, 2013](#_ENREF_5); [Hohenlohe *et al*, 2013](#_ENREF_7)) were obtained for use in this analysis (details of the sequences from each study are given in Table 5.1). To obtain consensus sequences across all populations, a custom-written clustering pipeline was applied. First, sequences across all four populations were combined into a single file (total number of sequences: 407,332). A BLASTN nucleotide database of all sequences in this file was created, and all sequences were aligned (BLASTN) to this database (i.e. self-alignment) [BLASTN version 2.2.25+, ([Altschul *et al*, 1990](#_ENREF_1))]. Alignments were quality filtered to retain only those with a inferred based on a minimum percentage identity of 95 %, and ≤ 2 base mismatch.

Homologous cross-population RAD loci were recovered as follows. For each sequence, the top match within each population for that query sequence was identified. For example, SEQ1_POP1 would align first to itself, then potentially to SEQX_POP2, SEQY_POP3 and SEQZ_POP4, and these were assigned to a single common RAD locus cluster. To reduce the inclusion of repetitive elements, sequences with high quality alignments to multiple clusters were removed, as were the clusters which they belonged to. Finally, clusters were filtered to retain those with a minimum of three and a maximum of four sequences. A total of 32,027 clusters were identified. For each cluster, a representative sequence was obtained, and this was used in all downstream analyses.

The Python script used to conduct this analysis is given below.

__author__ **=** 'Serap_Gonen'

# START DATE: 20/03/14

# END DATE: 20/03/14

# SCRIPT DESCRIPTION

# script was written to group rainbow trout loci across the four

# populations of RAD data

# criteria for grouping:

# > 95% identity

# < 3 mismatches

# criteria for a valid locus:

# must have at least 3 population matches (including

# itself) so is allowed to not match one population

# out of four

# INPUT FILE FORMAT:

# blastn -outfmt 7 output file (tab del)

# Fields: query id, subject id, query length, subject length,

# % identity, alignment length, mismatches, gap opens,

# q. start, q. end, s. start, s. end, evalue, bit score

# OUTPUT FILE FORMAT:

# Each row contains IDs of matched loci, tab del

# HOW TO RUN SCRIPT:

# Requirements: Python 2.6 and above. Not compatible with python3

# : Libraries : optparse

# Run on the command line as:

# python rtrout_common_loci_post_blastn.py

# --blastn_file <input_file>

#################################################################

# SCRIPT

#################################################################

# NECESSARY IMPORTS

# if script is main script being run

**if** __name__ **==** "__main__"**:**

# use optparse to specify input file

**from** optparse **import** OptionParser

parser **=** OptionParser**()**

parser**.**add_option**(**"--blastn_file"**,** dest **=** "blastn_file"**)**

**(**options**,** args**)** **=** parser**.**parse_args**()**

#################################################################

# OBJECT ORIENTED CODE

# class Locus

**class** **Locus(**object**):**

**def** __init__**(**self**):**

# holds the hits assigned to this locus

self**.**hit_list **=** **[]**

# assures that only one seq per pop is assigned to this locus

self**.**populations **=** **[**"MILLER"**,** "HOHENLOHE"**,** "HECHT"**,** "HALE"**]**

# method addHit

# adds subject_id to hit_list if a hit for that population hasn't

# been assigned before

**def** addHit**(**self**,** subject_id**):**

**for** population **in** self**.**populations**:**

**if** population **in** subject_id**:**

self**.**populations**.**remove**(**population**)**

self**.**hit_list**.**append**(**subject_id**)**

# class FileParser

**class** **FileParser(**object**):**

**def** __init__**(**self**):**

self**.**blastn_file **=\**

open**(**options**.**blastn_file**,**'r'**).**read**().**splitlines**()**

# keeps track of queries/subjects already assigned to a locus

self**.**seen **=** **[]**

# locus object dictionary

self**.**query2locusobj **=** **{}**

# method assignHit

# checks how good alignment is

# if good then sends subject to be assigned to locus

# if a hit for that population has not

# been assigned to that locus

# then checks if that subject_id has been assigned to the locus

# if it has then it adds it to the self.seen list

**def** assignHit**(**self**,** locus**,** subject_id**,** percentage_identity**,**\

mismatches**):**

**if** subject_id **not** **in** self**.**seen**:**

**if** float**(**percentage_identity**)** **>** 95.0 **and** \

int**(**mismatches**)** **<** 3**:**

locus**.**addHit**(**subject_id**)**

**if** subject_id **in** locus**.**hit_list**:**

self**.**seen**.**append**(**subject_id**)**

# method parseLines

# to actually read the file

**def** parseLines**(**self**):**

**for** line **in** self**.**blastn_file**:**

# split line into columns

query_id**,** subject_id**,** query_length**,** subject_length**,** \

percentage_identity**,** alignment_length**,** mismatches**,** \

gap_opens**,** q_start**,** q_end**,** s_start**,** s_end**,** evalue**,** \

bit_score **=** line**.**split**(**"\t"**)**

# if this is a new query

**if** query_id **not** **in** self**.**seen**:**

# make a new locus object

locus **=** Locus**()**

locus**.**addHit**(**query_id**)**

# say that we have now seen the query_id

self**.**seen**.**append**(**query_id**)**

# see if subject_id belongs to this locus

self**.**assignHit**(**locus**,** subject_id**,** \

percentage_identity**,** mismatches**)**

# add locus to the dictionary

self**.**query2locusobj**[**query_id**]** **=** locus

# if we have seen query_id and it is actually a locus

**elif** query_id **in** self**.**query2locusobj**.**keys**():**

# get the object

locus **=** self**.**query2locusobj**[**query_id**]**

# see if subject_id belongs to this locus

self**.**assignHit**(**locus**,** subject_id**,** \

percentage_identity**,** mismatches**)**

# else we have seen this query_id, however we saw it

# as a subject_id and not a query id

# class Combiner

**class** **Combiner(**object**):**

**def** __init__**(**self**):**

self**.**file_object **=** FileParser**()**

**def** runScript**(**self**):**

self**.**file_object**.**parseLines**()**

# print hits!

**for** locusobj **in** self**.**file_object**.**query2locusobj**.**values**():**

# locus seen in > 2 populations (ie 3, one of

# which is a match to itself)

**if** len**(**locusobj**.**hit_list**)** **>** 2**:**

**print** "\t"**.**join**(**locusobj**.**hit_list**)**

####################################################################

# PROCEDURAL CODE

Combiner**().**runScript**()**

# DONE

####################################################################

## Atlantic salmon

Two sets of RAD sequences were obtained from two different Atlantic salmon populations. The first set [SET1, ([Houston *et al*, 2012](#_ENREF_8))] was from a single-end RAD sequencing study conducted in two families [labelled as B and C in [Houston *et al* (2012](#_ENREF_8))], where RAD loci had been inferred separately within each family. Therefore, the first step in this analysis was the identification of common RAD loci across the two families. First, a BLASTN nucleotide database of the 337,315 RAD loci identified in family C was created. The 559,823 RAD locus sequences identified in family B were aligned (BLASTN) to this database. Alignments were filtered to retain those with high quality, based on a minimum percentage identity of 95 %, ≤ 2 base mismatch, and an E-value of 1e^-30^. These thresholds were determined by preliminary BLASTN alignments using simulated sequences of 95 base pairs (bp) in length, since this was the length of the sequences in both families. To eliminate RAD loci originating from repetitive regions, alignments where one or both of the sequences showed significant alignment to multiple sequences were removed. The final number of common RAD loci across the two families was 66,073.

The second set of RAD sequences [SET2, ([Gonen *et al*, 2014](#_ENREF_2))] was derived from paired-end RAD-sequencing, and was a mixture of 366,219 single- and 116,328 paired-end sequences (total: 482,547). For the purposes of this study, only the single-end sequences were utilised. A BLASTN nucleotide database of these sequences was created, and the 66,073 representative sequences from SET1 were aligned (BLASTN) to this database. As above, alignment significance was determined based on a minimum percentage identity of 95 %, ≤ 2 base mismatch, and an E-value of 1e^-30^, and filtering for RAD locus clusters originating from putative repetitive/duplicate regions of the genome was conducted based on the identification and removal of clusters containing sequences which mapped to multiple clusters. A total of 65,758 (99.5 %) shared RAD loci were identified across the two sets.

The Python script for processing of the resulting BLASTN file is given below:

__author__ **=** 'Serap_Gonen'

# START DATE: 10/03/14

# END DATE: 10/03/14

# SCRIPT DESCRIPTION

# Script to identify the best hits using a blast -outfmt 7

# output tabular file

# For matching within population - to get 1:1 correspondence

# between loci

# the following unix command would have already been run on the

# blastn tabular output file:

# cat FamilyB_vs_C.blastn | grep "^AS" | cut -f 1,2,11 | \

# awk 'BEGIN {FS = "\t"; query = ""}; {if($1!=query) \

# {print $1"\t"$2"\t"$3; query=$1} else{}} ' | sort -k2 > \

# query_hit_evalue.txt

# INPUT FILE FORMAT:

# query \t hit \t evalue

# OUTPUT FILE FORMAT:

# loci_from_file_1 \t loci_from_file_2

# HOW TO RUN SCRIPT:

# Requirements: Python 2.6 and above. Not compatible with python3

# : Libraries

####################################################################

# SCRIPT

####################################################################

# NECESSARY IMPORTS

**from** sys **import** argv

script**,** blastn_file **=** argv

####################################################################

# OBJECT ORIENTED CODE

**class** **FileParser(**object**):**

**def** __init__**(**self**,** blastn_file**):**

self**.**blastn_file **=** open**(**blastn_file**,** 'r'**).**read**().**splitlines**()**

self**.**hit2evalue **=** **{}**

self**.**hit2query **=** **{}**

**for** hit**,**query **in** self**.**hit2query**.**items**():**

**print** "{q}\t{h}"**.**format**(**q**=**query**,** h**=**hit**)**

**def** parseLines**(**self**):**

**for** line **in** self**.**blastn_file**:**

query**,** hit**,** evalue **=** line**.**split**(**"\t"**)**

**if** hit **not** **in** self**.**hit2evalue**:**

self**.**hit2evalue**[**hit**]** **=** evalue

self**.**hit2query**[**hit**]** **=** query

**else:**

last_evalue **=** self**.**hit2evalue**[**hit**]**

**if** float**(**last_evalue**)** **>** float**(**evalue**):**

self**.**hit2evalue**[**hit**]** **=** evalue

self**.**hit2query**[**hit**]** **=** query

######################################################################

# PROCEDURAL CODE

FileParser**(**blastn_file**).**parseLines**()**

# DONE

######################################################################

## Three-spined stickleback

Sequences from 46 stickleback originating from populations in Vancouver Island, British Columbia, Canada were kindly donated for this study by Dr Daniel Berner (Universität Basel, Zoologisches Institut, Switzerland) ([Roesti *et al*, 2012](#_ENREF_9); [Roesti *et al*, 2013](#_ENREF_10)). Since sequences originated from two independent sequencing experiments/technologies, read lengths across individuals were different, whereby ten individuals had sequence lengths of 138 bp, and the remaining 36 had sequence lengths of 64 bp. The number of sequences across all individuals ranged from 25,840 – 42,618.

Sequences across all individuals were combined into a single FASTA file containing 1,668,843 sequences. A BLASTN nucleotide database of this file was produced and aligned (BLASTN) to itself. Alignments were quality filtered, based on a minimum of 95 % match identity, maximum of 2 mismatches and alignment length (minimum of 64 bp if analysing the shorter reads, 138 bp otherwise). Filtered alignments were clustered into common RAD loci across individuals. If a single sequence was significantly mapped to multiple different clusters, this sequence, and the clusters it was assigned to, were removed from further analyses. The remaining clusters containing uniquely assigned sequences were filtered to retain those with a minimum of 20 sequences from 20 different individuals and a maximum of 50 sequences overall (to filter for repeats). A total of 31,118 clusters (i.e. shared RAD loci) were identified. A single representative sequence was selected and used in all downstream analyses.

The Python script used to implement this clustering pipeline is given below:

__author__ **=** 'Serap_Gonen'

# START DATE: 13/03/14

# END DATE: 15/03/14

# SCRIPT DESCRIPTION

# this script was written to parse blastn tabular output

# written specifically for the stickleback RAD data in

# order to match RAD loci between the 46 individuals

# however it can of course be adapted to other blastn comparsions

# and output, just by changing a few things

# INPUT FILE FORMAT:

# blastn output file in -outfmt 7 format. Columns:

# query id, subject id, % identity, alignment length,

# mismatches, gap opens, q. start, q. end, s. start, s. end,

# evalue, bit score

# OUTPUT FILE FORMAT:

# Locus 1

# defline of matching sequences as a column

# Locus 2

# defline of matching sequences as a column

# etc....

# HOW TO RUN SCRIPT:

# Requirements: Python 2.6 and above. Not compatible with python3

# : Libraries: optparse

# Run on the command line as:

# python blastn_output_parser.py

# --blastn_input all_46_individuals.blastn

# --max_num_hits 50

# --min_num_hits 20

# --percentage_identity 100

# --alignment_length 64,138

# --max_num_mismatches 2

# > common_loci.txt

##################################################################

# SCRIPT

##################################################################

# OBJECT ORIENTED CODE

**class** **CheckLine(**object**):**

**def** __init__**(**self**,** line**):**

self**.**line **=** line

**def** check**(**self**):**

**if** "hits" **in** self**.**line**:**

number_of_hits **=** int**(**self**.**line**.**split**(**" "**)[**1**])**

**if** number_of_hits **>** int**(**options**.**max_num_hits**)** **or** \

number_of_hits **<** int**(**options**.**min_num_hits**):**

**return** **False**

**else:**

**return** **True**

**class** **Locus(**object**):**

**def** __init__**(**self**,** name**):**

self**.**name **=** name

self**.**hit_list **=** **[]**

**def** addHit**(**self**,** hit**):**

self**.**hit_list**.**append**(**hit**)**

**def** validLocus**(**self**):**

**if** len**(**self**.**hit_list**)** **>** int**(**options**.**min_num_hits**):**

**return** **True**

**class** **AlignmentType(**object**):**

**def** __init__**(**self**,** percentage_identity**,** mismatches**,** \

alignment_length**):**

self**.**percentage_identity **=** percentage_identity

self**.**mismatches **=** mismatches

self**.**alignment_length **=** alignment_length

**def** perfect**(**self**):**

**if** float**(**self**.**percentage_identity**)** **==** \

float**(**options**.**percentage_identity**)** \

**and** self**.**mismatches **==** "0" \

**and** self**.**alignment_length **in** \

options**.**alignment_length**.**split**(**","**):**

**return** **True**

**else:**

**return** **False**

**def** good**(**self**):**

**if** int**(**self**.**mismatches**)** **<** 3**:**

**return** **True**

**else:**

**return** **False**

**class** **FileParser(**object**):**

**def** __init__**(**self**):**

self**.**blastn_input **=** open**(**options**.**blastn_input**,** \

'r'**).**read**().**splitlines**()**

self**.**locus_dict **=** **{}**

self**.**seen **=** **[]**

self**.**mismatch1_hit2query **=** **{}**

self**.**mismatch2_hit2query **=** **{}**

**def** updateMismatches**(**self**,** subject_id**,** query_id**,** mm_count**):**

mm_dict **=** **False**

**if** mm_count **==** "1"**:**

mm_dict **=** self**.**mismatch1_hit2query

**elif** mm_count **==** "2"**:**

mm_dict **=** self**.**mismatch2_hit2query

**if** mm_dict**:**

**if** subject_id **not** **in** mm_dict**:**

mm_dict**[**subject_id**]** **=** **[]**

mm_dict**[**subject_id**].**append**(**query_id**)**

**def** updateLocusDict**(**self**,** locus**):**

**if** locus**.**name **not** **in** self**.**locus_dict**.**keys**():**

self**.**locus_dict**[**locus**.**name**]** **=** locus

**def** getLocusObject**(**self**,** query_id**):**

**if** query_id **not** **in** self**.**seen**:**

self**.**seen**.**append**(**query_id**)**

**return** Locus**(**query_id**)**

**elif** query_id **in** self**.**locus_dict**.**keys**():**

**return** self**.**locus_dict**[**query_id**]**

**else:**

**return** **False**

**def** lineParser**(**self**):**

want **=** **False**

**for** line **in** self**.**blastn_input**:**

**if** line**.**startswith**(**"#"**):**

want **=** CheckLine**(**line**).**check**()**

**else:**

**if** want**:**

query_id**,** subject_id**,** percentage_identity**,\**

alignment_length**,** mismatches**,** gap_opens**,** \

query_start**,** query_end**,** subject_start**,** \

subject_end**,** evalue**,** bitscore **=** \

line**.**split**(**"\t"**)**

locus **=** self**.**getLocusObject**(**query_id**)**

**if** locus**:**

**if** subject_id **not** **in** self**.**seen**:**

alignment **=** \

AlignmentType**(**percentage_identity**,** \

mismatches**,** alignment_length**)**

**if** alignment**.**perfect**():**

locus**.**addHit**(**subject_id**)**

self**.**seen**.**append**(**subject_id**)**

**elif** alignment**.**good**():**

self**.**updateMismatches**( \**

subject_id**,** query_id**,** \

mismatches**)**

self**.**updateLocusDict**(**locus**)**

**class** **AssignMismatches(**object**):**

**def** __init__**(**self**,** locus_dict**,** mismatch_dict**,** seen**):**

self**.**locus_dict **=** locus_dict

self**.**mismatch_dict **=** mismatch_dict

self**.**seen **=** seen

**def** addMismatchHits**(**self**):**

**for** hit**,** query_list **in** self**.**mismatch_dict**:**

**if** len**(**query_list**)** **==** 1**:**

**if** hit **not** **in** self**.**seen**:**

self**.**locus_dict**[**query_list**[**0**]].**addHit**(**hit**)**

self**.**seen**.**append**(**hit**)**

**class** **TrueLocus(**object**):**

**def** __init__**(**self**,** locus_dict**):**

self**.**locus_dict **=** locus_dict

**def** locusParser**(**self**):**

**for** locus_id**,** locus_object **in** self**.**locus_dict**.**items**():**

**if** locus_object**.**validLocus**():**

**print** locus_id**,** "\t"**,** \

'\t'**.**join**(**locus_object**.**hit_list**)**

**class** **Combiner(**object**):**

**def** __init__**(**self**):**

self**.**file_object **=** FileParser**()**

**def** parseMismatches**(**self**):**

self**.**locus_dict **=** self**.**file_object**.**locus_dict

self**.**mismatch1_hit2query **=** \

self**.**file_object**.**mismatch1_hit2query

self**.**mismatch2_hit2query **= \**

self**.**file_object**.**mismatch2_hit2query

AssignMismatches**(**self**.**locus_dict**,** \

self**.**mismatch1_hit2query**,** self**.**file_object**.**seen**)**

AssignMismatches**(**self**.**locus_dict**,** \

self**.**mismatch2_hit2query**,** self**.**file_object**.**seen**)**

**def** printValidLoci**(**self**):**

TrueLocus**(**self**.**locus_dict**).**locusParser**()**

**def** scriptRunner**(**self**):**

self**.**file_object**.**lineParser**()**

self**.**parseMismatches**()**

self**.**printValidLoci**()**

####################################################################

# PROCEDURAL CODE

Combiner**().**scriptRunner**()**

# DONE

####################################################################

# References

Altschul SF, Gish W Fau - Miller W, Miller W Fau - Myers EW, Myers Ew Fau - Lipman DJ, Lipman DJ (1990). Basic local alignment search tool. *Journal of Molecular Biology* 215**:** 403-410.

Gonen S, Lowe NR, Cezard T, Gharbi K, Bishop SC, Houston RD (2014). Linkage maps of the Atlantic salmon (*Salmo salar*) genome derived from RAD sequencing. *BMC Genomics* 15**:** 166.

Hale MC, Thrower FP, Berntson EA, Miller MR, Nichols KM (2013). Evaluating adaptive divergence between migratory and nonmigratory ecotypes of a Salmonid fish, *Oncorhynchus mykiss*. *G3-Genes Genomes Genet* 3**:** 1273-1285.

Hecht BC, Thrower FP, Hale MC, Miller MR, Nichols KM (2012). Genetic architecture of migration-related traits in rainbow and steelhead trout, *Oncorhynchus mykiss*. *G3-Genes Genomes Genet* 2**:** 1113-1127.

Hecht BC, Campbell NR, Holecek DE, Narum SR (2013). Genome-wide association reveals genetic basis for the propensity to migrate in wild populations of rainbow and steelhead trout. *Mol Ecol* 22**:** 3061-3076.

Hohenlohe PA, Amish SJ, Catchen JM, Allendorf FW, Luikart G (2011). Next-generation RAD sequencing identifies thousands of SNPs for assessing hybridization between rainbow and westslope cutthroat trout. *Mol Ecol Resour* 11**:** 117-122.

Hohenlohe PA, Day MD, Amish SJ, Miller MR, Kamps-Hughes N, Boyer MC *et al* (2013). Genomic patterns of introgression in rainbow and westslope cutthroat trout illuminated by overlapping paired-end RAD sequencing. *Mol Ecol* 22**:** 3002-3013.

Houston RD, Davey JW, Bishop SC, Lowe NR, Mota-Velasco JC, Hamilton A *et al* (2012). Characterisation of QTL-linked and genome-wide restriction site-associated DNA (RAD) markers in farmed Atlantic salmon. *BMC Genomics* 13.

Roesti M, Hendry AP, Salzburger W, Berner D (2012). Genome divergence during evolutionary diversification as revealed in replicate lake-stream stickleback population pairs. *Mol Ecol* 21**:** 2852-2862.

Roesti M, Moser D, Berner D (2013). Recombination in the threespine stickleback genome patterns and consequences. *Mol Ecol* 22**:** 3014-3027.
