# Supplementary material for: Exploring the utility of cross-laboratory RAD-sequencing datasets for phylogenetic analysis
Source: BMC Res Notes. 2015 Jul 8;8:299. doi: 10.1186/s13104-015-1261-2 (PMC4495686; doi:10.1186/s13104-015-1261-2)
Supplement: Additional file 2: — Clustering of pairwise BLASTN alignments into cross-species orthologous RAD loci. [file 13104_2015_1261_MOESM2_ESM.docx]

# Clustering of pairwise BLASTN alignments into cross-species orthologous RAD loci

Pairwise BLASTN alignments were clustered into cross-species orthologous RAD loci as follows.

1. Identify ‘best hits’ for each pairwise alignment.
2. Filter best hits to identify only unique top alignments (i.e., one-to-one alignments)
3. Filter best hits based on sequence similarity parameters:
   1. Strict analysis, within salmonid species only
      1. 95 % sequence similarity
      2. ≤ 2 base mismatch
      3. Minimum 50 bp alignment
   2. Relaxed analysis, across all ten species
      1. 85 % sequence similarity
      2. ≤ 10 base mismatch
      3. Minimum 45 bp alignment
4. Generate a concatenated file of all filtered pairwise alignments across all species.
5. Group pairwise alignments into putative RAD clusters. E.g. within salmonid species only, if Atlantic_salmon_RAD_1 significantly aligned to Sockeye_salmon_RAD_1, Chinook_salmon_RAD_1, Lake_whitefish_RAD_1 and Rainbow_trout_RAD_1, and these all aligned to each other respectively, then these were inferred as a single cluster. Python script written for this is given below.
6. Identify sequences assigned to more than one cluster. Remove all clusters containing these sequences.
7. Filter clusters to remove those with more than one sequence originating from a given species.
8. Filter clusters for a minimum number of species sequences (E.g. minimum of 7 of the 10 species must have sequence etc.).
9. In the across teleost sprecies analysis, identify and remove salmonid-specific clusters.

__author__ **=** 'Serap_Gonen'

# START DATE: 07/05/14

# END DATE: 07/05/14

# SCRIPT DESCRIPTION

# This script uses blastn one to one matches to

# assign groups of loci to clusters, so that common # loci can be identified based on pairwise

# alignments

# INPUT FILE FORMAT:

# two input files:

# 1) blastn onetoone match file

# number of rows is irrelevant as long as first

# and second column are query and subject ID

# 2) single column file of sequences aligning

# to multiple(2/3/4 therefore present in 3/4/5)

# populations

# OUTPUT FILE FORMAT:

# sequence_id\tcluster

# HOW TO RUN SCRIPT:

# Requirements: Python 2.6 and above.

# : Not compatible with python3

# : Libraries : argv only (sys)

# Run on the command line as:

# python identify_common_loci.py \

# <blastn_file> <match_file> > <outfile>

####################################################

# SCRIPT

####################################################

# NECESSARY IMPORTS

**from** sys **import** argv

script**,** blastn_file**,** match_file **=** argv

####################################################

# PROCEDURAL CODE

# file of sequence headers matching across multiple

# pops

match_list **=** open**(**match_file**,** 'r'**).**read**().**splitlines**()**

# file of blastn one to one matches

blastn_list **=** open**(**blastn_file**,** 'r'**).**read**().**splitlines**()**

# dict for storing sequence id to the allocated cluster

sequence2cluster **=** **{}**

count **=** 0 # count to assign new cluster number

**for** line **in** blastn_list**:**

elements **=** line**.**split**(**"\t"**)**

**if** **(**elements**[**0**]** **in** match_list**)** **and** \

**(**elements**[**1**]** **in** match_list**):**

# elements[0]==query, elements[1]==sequence

**if** elements**[**0**]** **not** **in** sequence2cluster**:**

**if** elements**[**1**]** **not** **in** sequence2cluster**:**

# new rad locus, assign new cluster

sequence2cluster**[**elements**[**0**]]** **=** count

sequence2cluster**[**elements**[**1**]]** **=** count

count **+=** 1 # increment for new cluster

**else:**

sequence2cluster**[**elements**[**0**]]** **=** \

sequence2cluster**[**elements**[**1**]]**

**else:**

**if** elements**[**1**]** **not** **in** sequence2cluster**:**

sequence2cluster**[**elements**[**1**]]** **=** \

sequence2cluster**[**elements**[**0**]]**

# print

**for** sequence**,** cluster **in** sequence2cluster**.**items**():**

**print** sequence**,** "\t"**,** cluster

# DONE
