# Supplementary material for: Exploring the utility of cross-laboratory RAD-sequencing datasets for phylogenetic analysis
Source: BMC Res Notes. 2015 Jul 8;8:299. doi: 10.1186/s13104-015-1261-2 (PMC4495686; doi:10.1186/s13104-015-1261-2)
Supplement: Additional file 3: — Number and percentage of shared RAD loci identified by pairwise BLASTN alignments. [file 13104_2015_1261_MOESM3_ESM.docx]

**Number and percentage of shared RAD loci identified by pairwise BLASTN alignments**

|  | **Chinook salmon** | **Sockeye salmon** | **Rainbow trout** | **Atlantic salmon** | **Lake whitefish** | **Three-spined stickleback** | **Atlantic halibut** | **Baltic sea herring** | **Spotted gar** | **Gudgeon** |
| --- | --- | --- | --- | --- | --- | --- | --- | --- | --- | --- |
| **Chinook salmon** | NA | 32,648 (52.4) | 18,606 (58.1) | 24,108 (38.7) | 16,615 (26.7) | 595 (1.9) | 792 (1.3) | 520 (0.8) | 271 (0.4) | 439 (1.0) |
| **Sockeye salmon** | 32,911 (52.9) | NA | 19,625 (61.3) | 25,028 (38.7) | 17,739 (27.5) | 567 (1.8) | 625 (1.0) | 349 (0.6) | 283 (0.4) | 313 (0.7) |
| **Rainbow trout** | 19,338 (60.4) | 16,970 (52.0) | NA | 15,393 (48.1) | 11,026 (34.4) | 317 (1.0) | 493 (1.5) | 331 (1.0) | 187 (0.6) | 226 (0.7) |
| **Atlantic salmon** | 25,935 (41.7) | 27,118 (42.0) | 16,670 (52.0) | NA | 19,457 (29.6) | 609 (2.0) | 892 (1.4) | 459 (0.7) | 413 (0.6) | 472 (1.1) |
| **Lake whitefish** | 15,579 (25.0) | 21,402 (33.1) | 13,433 (41.9) | 18,035 (27.4) | NA | 711 (2.3) | 894 (1.1) | 421 (0.7) | 361 (0.6) | 427 (1.0) |
| **Three-spined stickleback** | NA | NA | NA | NA | NA | NA | 2,704 (8.7) | 368 (1.2) | 197 (0.6) | 228 (0.7) |
| **Atlantic halibut** | NA | NA | NA | NA | NA | NA | NA | 310 (0.5) | 330 (0.5) | 362 (0.8) |
| **Baltic sea herring** | NA | NA | NA | NA | NA | NA | NA | NA | 213 (0.3) | 434 (1.0) |
| **Spotted gar** | NA | NA | NA | NA | NA | NA | NA | NA | NA | 199 (0.5) |
| **Gudgeon** | NA | NA | NA | NA | NA | NA | NA | NA | NA | NA |

Percentages (in parentheses) are given relative to the fish with the fewest number of sequences in processed FASTA files. Upper quadrant of matrix shows values obtained from the ‘relaxed’ analyses. The number and percentage of alignments obtained from the salmonid ‘strict’ analysis is given in the lower quadrant of the matrix.
