# Supplementary material for: Exploring the utility of cross-laboratory RAD-sequencing datasets for phylogenetic analysis
Source: BMC Res Notes. 2015 Jul 8;8:299. doi: 10.1186/s13104-015-1261-2 (PMC4495686; doi:10.1186/s13104-015-1261-2)
Supplement: Additional file 4: — Parameters for phylogenetic tree construction using RAxML. [file 13104_2015_1261_MOESM4_ESM.docx]

# Parameters for phylogenetic tree construction using RAxML V 8.1.13

## RAxML

Reference: Stamatakis, A., 2014. RAxML Version 8: A tool for Phylogenetic Analysis and Post-Analysis of Large Phylogenies. *Bioinformatics*. **30**(9):1312–1313.

Version: RAxML 8.1.13, released by Alexandros Stamatakis on 16^th^ December 2014

The command line version of RAxML was used in this analysis. The steps for tree construction, and a brief description of input parameters, are given below.

Steps:

| **Step** | **Command** | **Output files** |
| --- | --- | --- |
| 1) Obtain most parsimonious tree from the input data | /path/to/raxml  -m ASC_GTRGAMMA  -p 123456  -s file_input.phylip  -n Tree1  -N 10000  -f o  --asc-corr=lewis  (-o outgroup) | a) One output file for each run  b) RAxML_bestTree.Tree1 file, which is the tree with the maximum likelihood, given the input data  c) RAxML_info.Tree1 file, with logs of output to terminals |
| 2) Bootstrap  to obtain estimates of tree confidence | /path/to/raxml  -m ASC_GTRGAMMA  -b 123456  -p 123456  -s file_input.phylip  -n Tree2  -N 10000  -f o  --asc-corr=lewis  -k  (-o outgroup) | a) RAxML_bootstrap.Tree2 which contains the values from the bootstrap runs  b) RAxML_info.Tree2 |
| 3) Check if a sufficient number of bootstraps were performed | /path/to/raxml  -m ASC_GTRGAMMA  -z RAxML_bootstrap.Tree2  -I autoMRE  -n Tree3  --asc-corr=lewis  -p 123456 | Screen output only |
| 4) Write tree support values from bootstrapping on to most parsimonious tree from step 1 | /path/to/raxml  -m ASC_GTRGAMMA  -p 123456  -t RAxML_bestTree.Tree1  -z RAxML_bootstrap.Tree2  -n Tree4  -N 10000  -f b  --asc-corr=lewis  (-o outgroup) | a) RAxML_bipartitions.Tree4 which contains the node supports  b) RAxML_bipartitionsBranchLabels.Tree4 which contains support values on nodes and branches |

Input parameter descriptions:

| **Parameter** | **Option** | **Description** |
| --- | --- | --- |
| -m | ASC_ GTRGAMMA | Model for estimating tree parameters |
| -p | 123456 | This can take any value. It is a way of ensuring that parameter estimations start from the same value (to make results reproducible) |
| -b | 123456 | Specifies the requirement for bootstrapping. This can take any value and allows reproducibility of runs using the input integer as a starting value |
| -f | b | Used to draw bipartitions on an input tree specified using -t, using the bootstrap tree parameters file specified in -z |
|  | o | Specifies use of older (and slower) algorithm to obtain log likelihoods. Estimates obtained using this algorithm are thought to be typically better |
| -N | 10000 | Executes 10,000 maximum likelihood searches, using 10,000 different starting trees |
| -n | TreeX | Output file name extension |
| -t RAxML_bestTree.Tree1 |  | Most parsimonious tree from the input data produced in step 1. A user specified tree can also be given as input using this parameter, resulting in bootstrap results from step two being used to obtain support for the user specified tree instead |
| -z RAxML_bootstrap.Tree2 |  | Output file with bootstrap statistics |
| --asc-corr | lewis | Standard Lewis correction for ascertainment bias correction due to use of between species variants |
| -k | N/A | Bootstrapped trees will be printed with branch lengths |
| -I | autoMRE | Option to check for bootstrap convergence in step 3 |
| -o outgroup |  | This option was used when specifying spotted gar as outgroup to the salmonids |

Further details for running analyses using RAxML can be found in the manual: <http://sco.h-its.org/exelixis/resource/download/NewManual.pdf>.
