# Supplementary material for: Exploring the utility of cross-laboratory RAD-sequencing datasets for phylogenetic analysis
Source: BMC Res Notes. 2015 Jul 8;8:299. doi: 10.1186/s13104-015-1261-2 (PMC4495686; doi:10.1186/s13104-015-1261-2)

# Estimated phylogenetic relationships

Evolutionary relationships amongst ten teleost fish species were reconstructed based on RAD-Seq data, using the RAxML software (version 8).

Trees were visualised using one of the three following software:

- Phylodendron (<http://iubio.bio.indiana.edu/treeapp/treeprint-form.html>)
- T-REX ([Boc et al, 2012](#_ENREF_33))
- Archaeopteryx ([Han and Zmasek, 2009](#_ENREF_134))

For each figure, branch lengths estimated by RAxML are given in red text along individual branches, and bootstrap node supports are given in green text at each node.


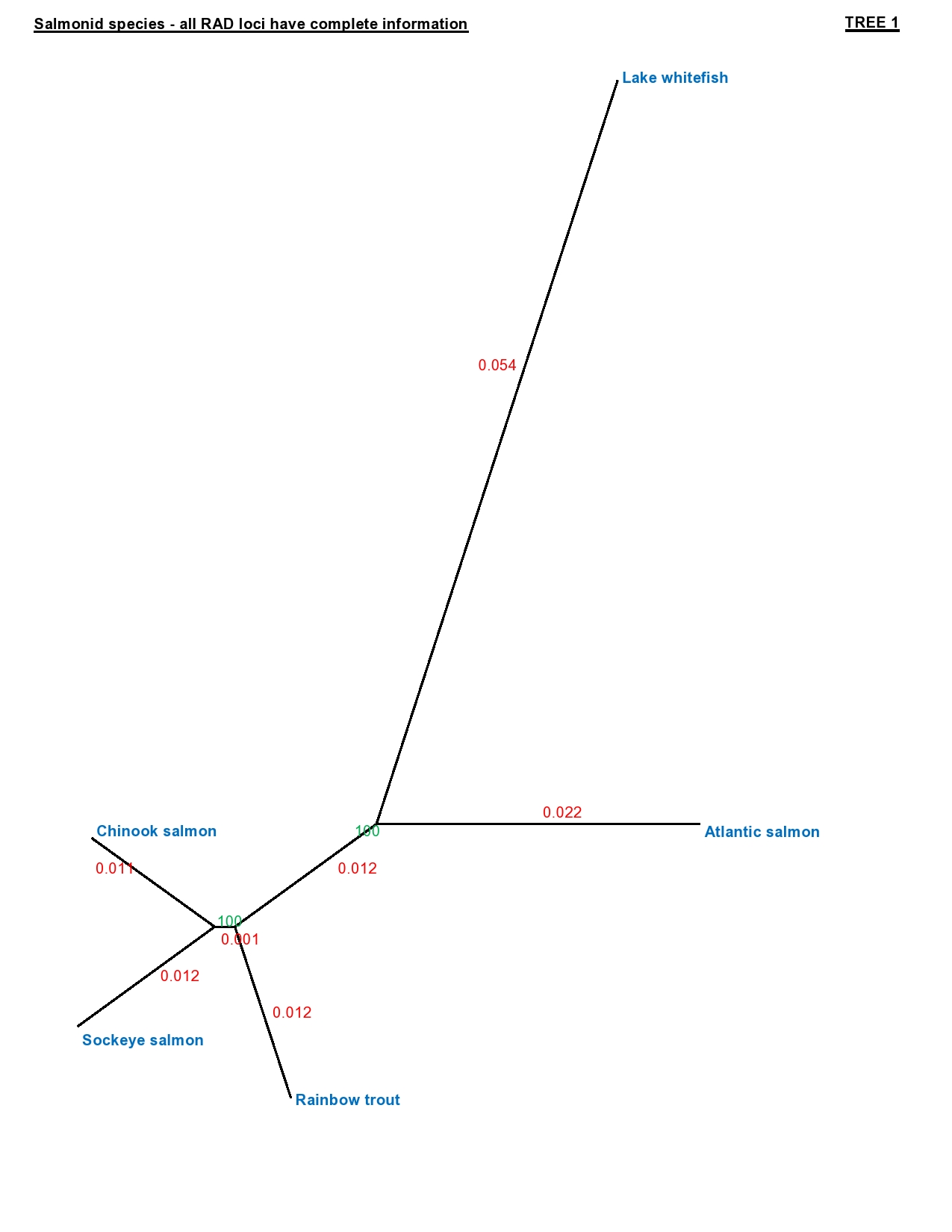


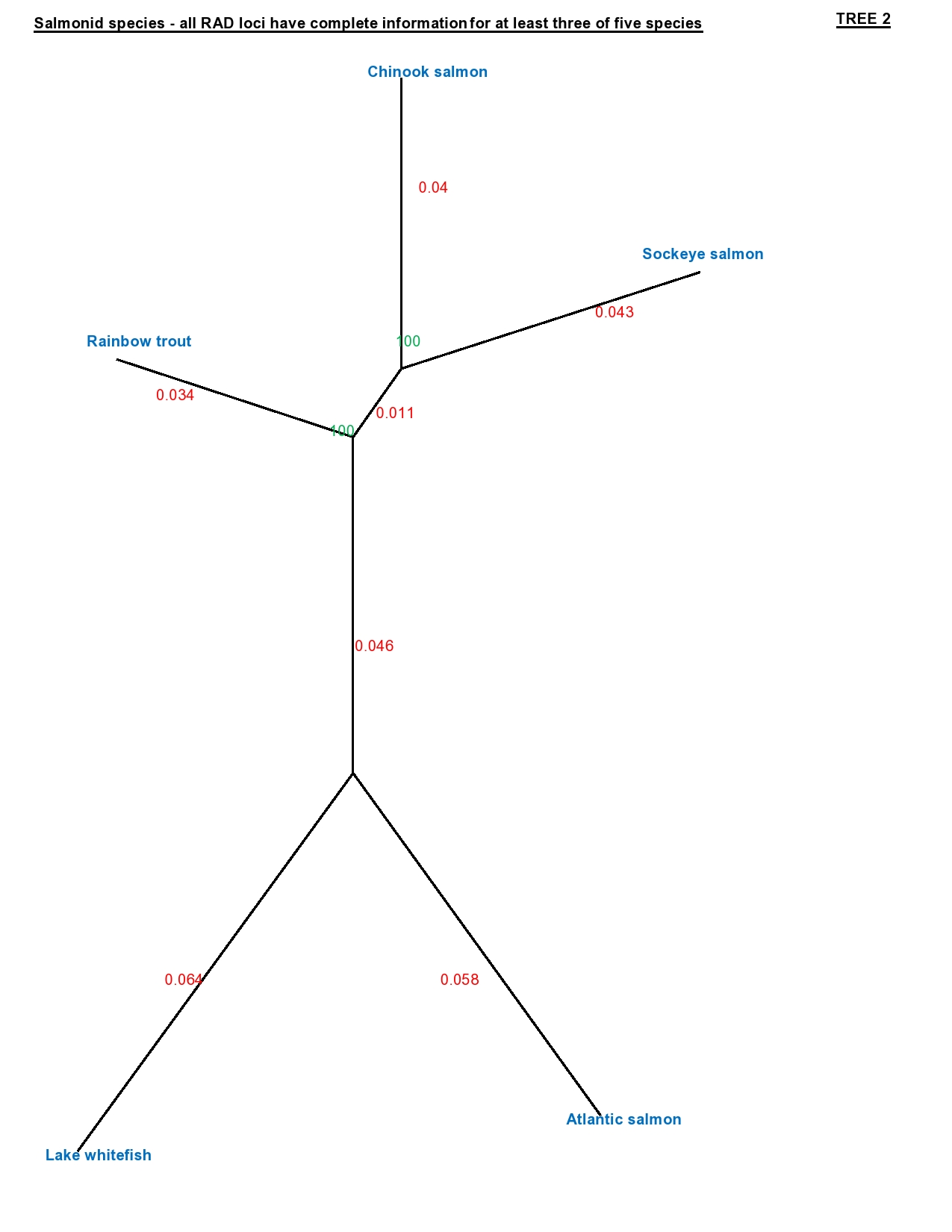


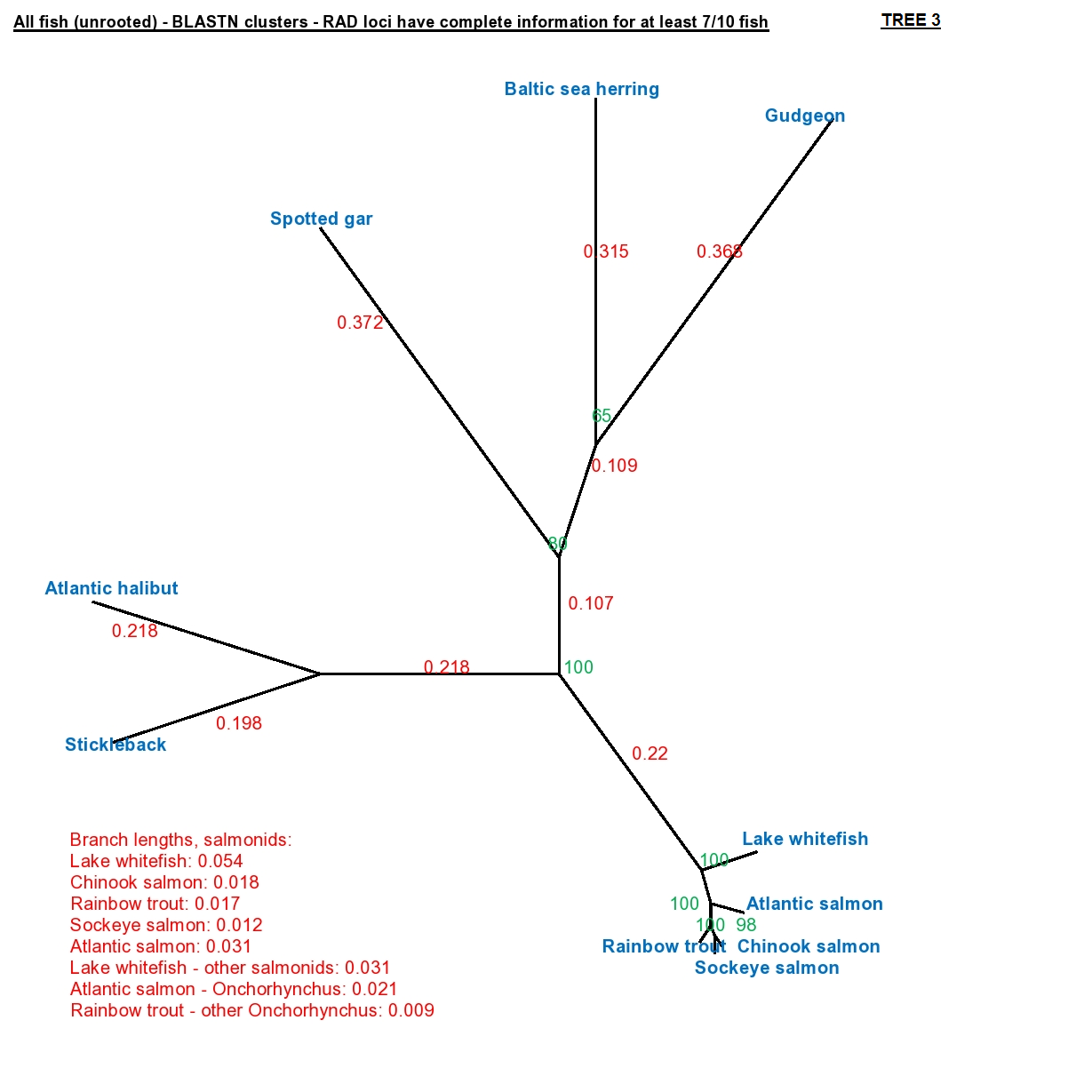


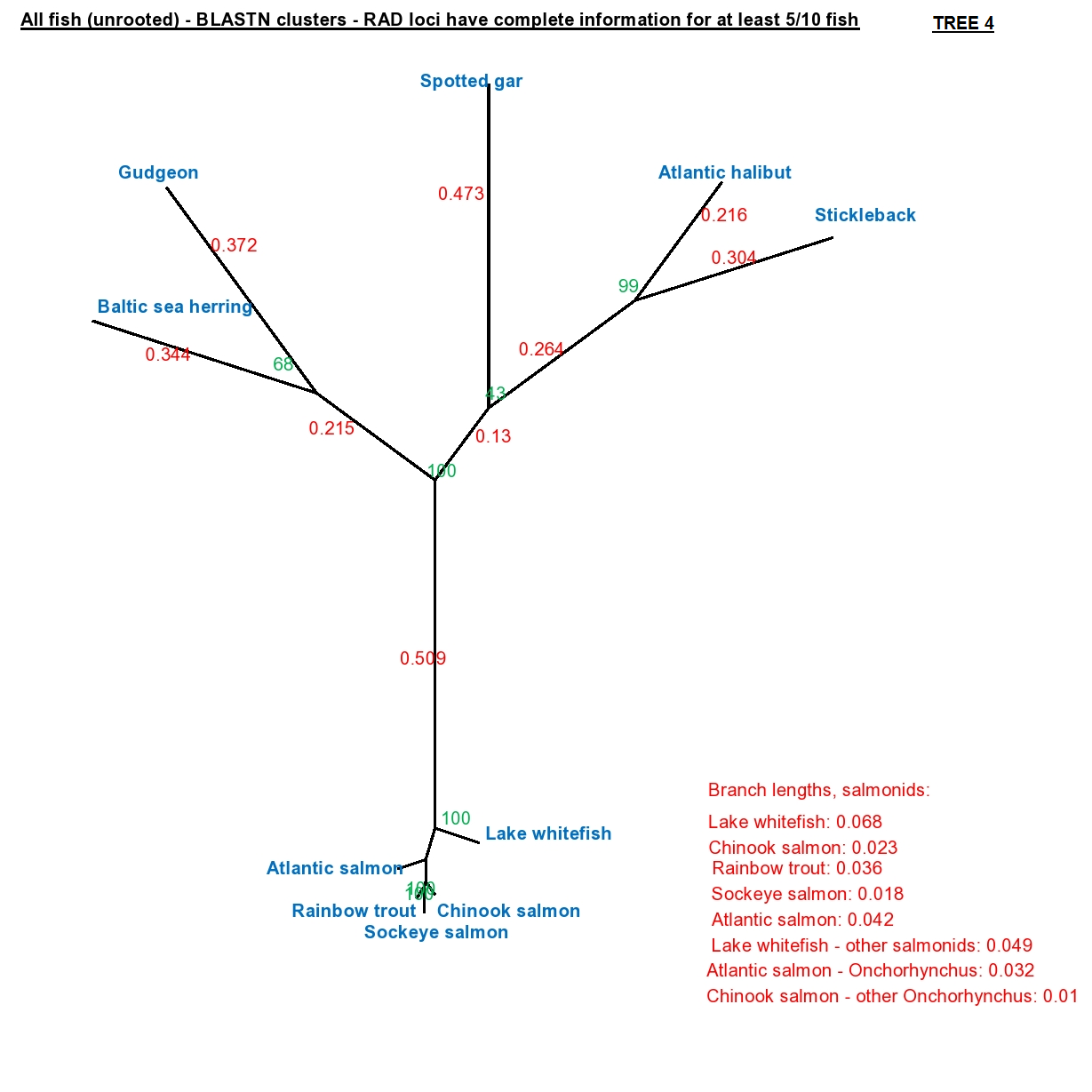

Supplement: Additional file 5: — Estimated phylogenetic relationships. [file 13104_2015_1261_MOESM5_ESM.docx]
